# Uncovering the Experiences of Basti-Dwelling Adolescent Girls Who Menstruate in Hyderabad, India: Protocol for a Multimethod Critical Qualitative Study

**DOI:** 10.2196/94012

**Published:** 2026-10-19

**Authors:** Snigdha Velugu, Erica Di Ruggiero, Nanda Kishore Kannuri, Anushka Ataullahjan

**Affiliations:** 1 Social and Behavioural Health Sciences Division, Dalla Lana School of Public Health, University of Toronto, 27 King's College Circle, Toronto, ON, M5S 1A1, Canada, 1 416-978-2011; 2 Department of Anthropology, University of Hyderabad, Hyderabad, Telangana, India; 3 School of Health Studies, Western University, London, ON, Canada

**Keywords:** menstruation, menstrual hygiene management, urban slums, India, post structuralist feminism, biopolitics, critical ethnography

## Abstract

**Background:**

Rapid urbanization in India is straining the government’s capacity to provide basic amenities such as housing, sanitation, electricity, and water. One group of people who are deeply affected are adolescent girls who menstruate. In urban slums, referred to as bastis in Telugu and Hindi, this reality is worsened by spatial congestion and limited water, sanitation, and hygiene (WASH) resources. This study is situated in the populous slum colonies of Film Nagar, Hyderabad, where residents navigate precarious living conditions and a scarcity of basic amenities.

**Objective:**

In this context, we argue that menstrual experiences are profoundly shaped by an interplay of biological, sociopolitical, and environmental factors. This study aims to examine how these intersecting dimensions shape the menstrual experiences of slum-dwelling adolescent girls (aged 8‐18 years) in Film Nagar by understanding the (1) sociocultural and political discourse, (2) intergenerational household power dynamics, and (3) the enabling environment.

**Methods:**

Guided by biopolitics and poststructuralist feminist theory, we use a critical ethnographic approach involving intergenerational households (adolescent girls, mothers, fathers, and unmarried brothers aged 15‐24 years). Approximately 18‐28 informants across 5‐8 households will be recruited using maximum variation, purposive, and snowball sampling, targeting. Data generation includes rapport-building, participant observation (journaling, voice recordings, photography, or transect walks), document analysis, focus group discussions (FGDs), and in-depth interviews (IDIs). Analysis occurs concurrently using frame analysis: transcripts undergo value or narrative coding to inductively identify codes, followed by axial and selective coding to group codes into discourses interpreted through our theoretical framework, yielding dominant frames addressing each research question. Findings will inform a digital storytelling output along with a 3-paper thesis and peer-reviewed journal publication.

**Results:**

This study is part of the primary author’s doctoral dissertation, funded by the Social Sciences and Humanities Research Council (SSHRC) Doctoral Fellowship (2023‐2026) and the International Development Research Centre (IDRC) International Doctoral Research Award (2024‐2025). Ethics approval was obtained from the University of Toronto and University of Hyderabad research ethics boards in January 2025. Recruitment closed in September 2025 with 18 households enrolled. Data collection closed in March 2026. Protocol deviations included narrowing the study from 5 to 2 slum colonies due to time constraints, resequencing methods to begin with maternal focus groups to build trust, shifting to small group discussions in participants’ homes, adding ethnographic interviews, and permitting verbal consent; 3 of these changes required Research Ethics Board (REB) amendment. Dissemination is anticipated by September 2027.

**Conclusions:**

This study is expected to (1) clarify how sociopolitical, familial, and environmental factors interact to shape menstrual experiences among adolescent girls in urban slums and (2) inform context-sensitive menstrual health policies and programs for similar low-resource settings.

## Introduction

### Background

In biomedical terms, menstruation is a biological phenomenon defined as the monthly shedding of the uterine lining of a nonpregnant woman. But menstruation is more than a biomedical experience. Menstruation is a vital sign of health that influences a woman’s physical, psychological, and reproductive well-being from puberty to menopause [1,2]. Menstruation and menstrual practices involve a complex interplay of sociocultural, political, historical, and environmental factors [3-5]. These contextual factors are influenced by discourses surrounding menstruation, the menstruating body, and its social role in bearing and raising children [4,6,7].

Menstrual discourses are known to have religious and cultural origins, from a time when people lacked the scientific knowledge to understand the physiological process of menstruation [8-10]. Major world religious texts, including those of Judaism, Christianity, Islam, Hinduism, and Buddhism, have historically used labels such as “impure,” “sick,” “dangerous,” and “sinful” to describe menstruation. These texts have also prescribed restrictions on physical intimacy, cooking, and attending places of worship while menstruating [8,9]. In India specifically, these restrictions have extended to practices such as prohibiting menstruating adolescent girls from bathing and requiring them to live separately from the household for the duration of their period [10]. To date, these dominant discourses are passed down intergenerationally and continue to dictate menstrual practices globally.

The implications of menstrual restrictions can vary. While for some, these restrictions can lead to discrimination and social isolation, some adolescent girls who menstruate enjoy this brief time off from their daily chores during their period, and a few others carry on with their daily lives irrespective of their period. The relevance and severity of these restrictions depend on one’s physical and social environment, which is defined by one’s access to menstrual infrastructure and period products such as toilets and sanitary pads, menstrual education, and the presence of a supportive social environment [5,11-14].

### Menstruation in the Urban Slums of India

To menstruate in an urban slum in India is to navigate lack of social support, financial hardship, and structural neglect leading to limited access to basic services such as housing, water, and electricity [3,11,15-17]. For instance, a typical household may consist of a single room of approximately 10 ×10 feet that serves as a bedroom at night and as a kitchen and living area by day, with narrow lanes as little as 5‐6 feet wide separating rows of houses [15]. Women may spend between 30 minutes and 2 hours each day collecting water from municipal tankers, and where this supply is insufficient, households sometimes rely on illegally constructed groundwater borewells [15]. Community toilets, where available, often charge a user fee, may be located 1‐2 kilometers away, and typically operate only between 6 AM and 10 PM, forcing residents to resort to open defecation at night [15]. These challenges are only going to increase in the coming decades. In India, where one-third of the population lives in cities, the slum-dwelling population is expected to rise by 41% by 2030 [15], consistent with broader projections that an estimated 60% of the world’s population will live in cities by 2030, with nearly 6.3 billion people living in urban areas by 2050 [11]. This rapid rate of urbanization in India is straining the government’s capacity to meet essential infrastructural and service needs [11]. The lack of infrastructure affects many in low-resource settings, but people who menstruate experience it differently. Unsafe environments, characterized by issues such as alcoholism, inadequate sanitation, and poorly maintained shared toilets—with problems like broken latches, missing doors, or insufficient lighting—expose women and girls to the risk of sexual abuse [11,18-20]. The scarcity of basic resources necessary for managing menstruation in comfort and safety can provoke communal tensions and moral scrutiny, sometimes leading to conflicts or reputational harm for families within the community [3,15]. The absence of space and privacy further exacerbates the situation. Without designated waste bins at toilet facilities, used menstrual materials are frequently discarded in open dumping areas, where they may be torn apart by stray animals, compounding both hygiene concerns and the risk of public exposure [15]. Moreover, waste disposal sites are often poorly maintained and infrequently cleaned by local municipal workers, compounding the challenges around sanitation and hygiene that are crucial during menstruation. Furthermore, in a slum setting, menstruation can be burdensome for both the adolescent girl and their families. The community’s awareness that a girl has reached menarche can be read as a sign of sexual maturity, drawing unwanted attention from men in the community. Combined with limited privacy and crowded living conditions, this can increase girls’ vulnerability to unwanted romantic or sexual advances, and in turn to unintended pregnancies through consensual relationships or sexual violence [3].

### Gaps in Menstrual Hygiene Management Research and Practice in India

Menstruation is often portrayed as an inconvenience. In advertisements, movies, and documentaries, and even in global menstrual health mandates, the primary focus is on improving menstrual hygiene and addressing menstrual knowledge and practices among adolescent girls in urban and rural settings, often linking these issues to school absenteeism [4,7,21]. For instance, in 2010, the Government of India launched the National Guidelines for Menstrual Hygiene Management (NGMHM), developed by the Water Supply and Sanitation Collaborative Council (WSSCC), that recommended a 3-pronged approach to improve menstrual hygiene management (MHM) in India: (1) breaking the silence, (2) managing menstruation safely with dignity, and (3) safe disposal [4]. But much of the work to improve MHM is singularly focused on distributing sanitary pads over improving a woman’s agency to care for their body with comfort, convenience, and choice [4].

Such a dominant focus on hygiene highlights significant gaps: a 2016 systematic review revealed that, compared to more than 50 studies in urban and rural areas, only 10 studies specifically addressed MHM in slum settings [22]. Moreover, the role of household power dynamics in shaping menstrual practices remains underexplored, even though menstrual health and hygiene are negotiated collectively within families and communities—not by adolescent girls alone.

The foregoing background on menstruation and menstrual experiences elucidates India’s approach to MHM, which is notably biomedical and significantly shaped by the prevailing MHM discourses surrounding hygiene, freedom, dignity, and productivity.

Slum-dwelling adolescent girls face challenges on a daily basis, such as negotiating privacy, respect, safety, and security. For instance, studies in low-resource areas of India have documented that women and girls often lack access to safe, private sanitation facilities, forcing them to manage menstruation in shared or unlit spaces, which heightens their vulnerability to sexual harassment and assault, particularly given broken latches or absent doors on shared toilets and poorly lit surroundings at night [11]. MHM initiatives, with their one-size-fits-all solutions for promoting and distributing sanitary pads, seem to ignore these unique contextual challenges, alongside others such as financial instability, lack of space, alcoholism, and inadequate sanitation, faced by slum-dwelling adolescent girls [11].

Rothchild and Piya [23] describe, within the Nepalese context, how discriminatory norms, beliefs, practices, and attitudes surrounding menstruation create a “complex web of control” that limits the agency and bodily autonomy of adolescent girls at a very young age. This can be translated to the Indian context, where similar stigmatizing menstrual beliefs and practices prevail. In low-resource settings specifically, these have been documented to include restrictions on sexual activity and participation in religious practices during menstruation, alongside a broader “culture of silence” that leaves many girls unprepared for and unable to openly discuss their first period [16]. Within the broader South Indian context, these restrictions have been documented in further concrete detail: girls have reported being barred from entering the “puja” (worship) room and temples, being prohibited from cooking for others, being segregated from other household members, using a separate eating plate, and being told to avoid contact with male family members, including their own fathers, with some describing this sudden imposed distance as distressing and isolating [24]. Even in research settings involving researchers and non-governmental organizations (NGOs), this taboo is so pronounced that women avoid or hesitate to participate in open group discussions on menstruation, highlighting the constrained mobility and social scrutiny women face in raising the topic at all [17]. These norms are shaped less by formal health education than by inherited sociocultural expectations and personal discretion within the household [16]. This form of control and silence restricts girls’ ability to make independent, informed decisions about their own bodies during menstruation [23]. Compounding this, the myopic and individualistic approach taken by the government and research agencies places the responsibility of menstrual health management solely on the person who menstruates. This perspective overlooks the crucial roles that both men and women within families and communities play in supporting adolescent girls.

Goddard and Sommer [11] emphasize the urgent need to explore the lived experiences of adolescent girls in slum environments. Drawing on their findings, menstrual experiences can be understood as shaped by an interplay of several factors. These factors include (1) vulnerability and risks associated with transitioning into adolescence, such as the risk of sexual violence linked to visible signs of sexual maturity; (2) sociocultural factors, including a pervasive culture of silence and traditions framing menstruation as impure; (3) political factors, such as the exclusion of informal slum settlements from municipal governance, data collection, and service provision; (4) economic factors, including access to water and sanitation infrastructure being contingent on the ability to pay rather than geographic proximity; and (5) environmental factors, such as the overcrowded and under-resourced living conditions produced by rapid, poorly managed urbanization [11]. To address the complex challenges faced by adolescent girls in these settings, our research seeks to comprehensively understand how these determinants manifest in everyday life shaping their menstrual experiences.

### Theoretical Framework

We take a critical ethnographic approach to understand the lived experiences of menstruators as shaped by dominant menstrual discourses. We draw on biopolitics [25] and poststructuralist feminism [20,26] to guide our methodological approach. Aligned with the critical social paradigm [27], these theories allow us to study the role of sociocultural and historical systems of power in constructing menstrual discourses that define menstrual experiences.

Biopolitics will assist in understanding the disciplinary techniques exercised by those in power to control and regulate adolescent girls’ bodies and conduct. These techniques help understand how institutionally driven prohygiene campaigns and policies exercise power to construct and regulate the “hygienic” menstruating body through self-surveillance mechanisms among adolescent girls. Concretely, this concept shapes our interview guides by prompting specific questions about girls’ and families’ encounters with menstrual norms, institutional actors, and messaging around menstruation. For example, questions about menstrual awareness and practices at home, school-based hygiene education, government sanitary pad distribution schemes, and instructions received from health workers or teachers, and how girls describe monitoring or correcting their own behavior in relation to this guidance. During coding, narrative coding [28] is used to identify instances where participants describe the family and institutionally derived rules being internalized as self-monitoring or self-correction (eg, checking for stains, timing bathroom use, and hiding products), which are treated as markers of biopolitics in the data.

Poststructural feminism, by highlighting the relationship between people and their sociocultural and historical contexts, helps elucidate how these disciplinary techniques get absorbed into family values, social norms, stigmatizing practices, and media representation. Furthermore, this theoretical framing helps explain how adolescent girls come to understand and experience their own menstruation through this sociocultural meaning-making. This concept correspondingly shapes our interview guides through questions probing girls’ and family members’ own language and explanations for menstrual restrictions—for example, asking why particular practices (eg, restrictions on cooking, worship, or mobility) are followed, who taught them, and how girls describe their own sense of self during menstruation. During coding, value coding [28] is used to identify the values, beliefs, and attitudes participants express about menstruation, which are treated as evidence of discursive subject formation—that is, how girls come to understand themselves as particular kinds of subjects (eg, “impure,” “grown up,” and “at risk”) through inherited language and norms rather than through institutional instruction alone.

Together, these theories allow us to understand how the discourses of menstruation promoted through various social control elements may complement, compete, or contradict each other in shaping menstrual experiences in slums. In practice, this dual lens is operationalized during axial and selective coding [28,29], where codes generated inductively (through value and narrative coding) are grouped into discourses and examined for whether they originate primarily from institutional hygiene-based messaging (biopolitics), inherited familial sociocultural norms (poststructuralist feminism), or reflect both the tension and interplay between the two. A dominant frame is considered to reflect this interplay when it recurs across both institutionally oriented data (eg, references to school or health worker instruction) and family or community-oriented data (eg, references to inherited practice or belief), consistent with the frame validation approach described in the Data Analysis section.

### Researcher Positionality

Between 2013 and 2015, the primary author of this paper taught at a public school through the Teach for India Fellowship, where many students lived, and she has maintained contact with several since. This community later became her PhD fieldwork site. This history initially suggested an insider-outsider position, but fieldwork revealed a substantial class distance. Although she was born and raised in the same city, and spoke the same language, she was raised by physician parents and was educated and employed across the United States and Canada; her mobility and lived experience of menstruation (unrestricted by school, sport, or space) differed sharply from her informants’. Fieldwork also corrected several of her assumptions, including that menstrual restriction from religious spaces was not generalized to all girls from conservative families. Rather, it was specific to Hindu, not Christian families. These moments shaped her approach: treating apparent similarity as a prompt for inquiry rather than an interpretive shortcut.

### Objectives and Research Questions

The objective of our proposed study is to explore how the interconnected environments and menstrual discourses shape the lived experiences of slum-dwelling adolescent girls who menstruate in Hyderabad, India.

To achieve this objective, we intend to answer the following questions:

1. Research question 1: How do adolescent girls who menstruate in urban slums perceive the sociocultural and political discourses shaping their menstrual experiences?
2. Research question 2: How do power dynamics in an intergenerational household of an adolescent girl who menstruates in slums shape their menstrual experiences?
3. Research question 3: How does the enabling environment influence a slum-dwelling adolescent girl’s menstrual experience?

## Methods

### Study Design

We use a multimethod critical qualitative design, incorporating participatory methodologies into critical ethnography (CE), to explore these dynamics among slum-dwelling adolescent girls in Film Nagar, Hyderabad. CE, referred to as “critical theory in action” and “traditional ethnography with a political agenda” [30], is particularly suited to this study’s theoretical framework, as it allows researchers to observe, critique, and reflect on how power operates through sociocultural, institutional, and material channels to shape menstrual norms and practices [30,31]. Data will be generated through rapport-building activities, participant observation (including journaling, transect walks, and photographic documentation), document analysis, and focus group discussions (FGDs) and in-depth interviews (IDIs) with intergenerational household members, including adolescent girls, mothers, fathers, and unmarried older male siblings.

### Research Setting and Population

The study will be conducted in the urban slum colonies of Film Nagar, Hyderabad, India. In India, the term “slum” refers to informal or under-resourced urban communities where low-income residents live in inadequate housing conditions and often have limited access to basic government services, regardless of whether they hold legal rights to the land [32]. These communities could be generations old or newer settlements. The slum colonies of Film Nagar are among the most populous slums in Hyderabad, India, struggling to provide their residents with basic living amenities. Despite being surrounded by an affluent community of film and media fraternities, politicians, and tech companies, a 2012 news report found that 80% of Jubilee Hills constituency’s population lived in various slum pockets dotting the area, with a particularly visible concentration of approximately 20 slum colonies along the Film Nagar–Shaikpet stretch [33]. Residents of these settlements are courted as the most sought-after vote bank in the constituency during election periods. A vote bank refers to a loyal bloc of voters, often organized along caste, religious, regional, or economic lines, who consistently support a particular political candidate or party in exchange for material or in-kind benefits promised or delivered before elections. The same 2012 report documented persistent neglect, thereafter. Unstable housing, acute water scarcity, poor health, hygiene, sanitation facilities, and drug and alcohol abuse are some of the key sources of tension faced by the residents of this slum battle daily, leading to community clashes [33,34].

To recruit participants, we will partner with Ishva Foundation, which has strong existing ties within the community. This foundation was first established eight years ago and runs an after-school program in the same slum and a few others across Hyderabad. With approval from the Research Ethics Board (REB) approval, we will draw our sample of informants from Ishva Foundation’s student registry and will use maximum variation purposive sampling [18,19] as well as snowball sampling. In this study, maximum variation sampling was conducted across the following variables: age, religion, caste, employment status, educational status of parents, and educational status of the adolescent menstruator. The use of both Ishva Foundation’s student registry and snowball sampling through community connectors served to operationalize this variation by capturing households both within and outside the formal school system, ensuring that participants with differing levels of institutional exposure, socioeconomic backgrounds, and cultural contexts were represented in the sample. This sampling approach will allow research informants to participate in the study at their discretion with no coercion [19]. Our sampling unit consists of intergenerational households that include adolescent girls who menstruate, their mothers, fathers, and, when applicable, unmarried male siblings aged 15‐24 years. Adolescent girls eligible for this study are between 8 and 18 years of age. Within this group, girls are further categorized based on time elapsed since menarche, rather than chronological age alone: (1) novice menstruators, defined as those who reached menarche 1‐2 years prior to the time of data collection, and (2) experienced menstruators, defined as those who reached menarche 3 or more years prior to the time of data collection and remain unmarried. For example, a girl who experienced menarche at age 8 years would be classified as a novice menstruator if interviewed within the following 1‐2 years, but as an experienced menstruator if interviewed 3 or more years after menarche, regardless of her age at the time of interview. This diverse representation is vital, as menstrual experiences are influenced by social, cultural, and physical contexts.

Unmarried male siblings aged 15‐24 years will be included to capture how an adolescent girl’s household environment, rather than her later marital environment, shapes her menstrual experience. Married men are excluded because they typically acquire menstrual knowledge primarily through their wives, a source formed outside the natal household this study examines. Restricting this category to unmarried siblings isolates household-of-origin influences on menstrual perception from spousal influences. The lower bound of 15 years reflects the age boys typically gain some household exposure to menstruation-related discussion, even though formal sexuality education in Indian schools is introduced only from class 9 onward and is often not fully imparted [35]. Boys are often deliberately excluded from menstruation-related content even when girls receive it, as the topic is considered not to concern them and remains taboo at home [28,29,36]. The upper bound of 24 years reflects the typical premarital age range for men in this population. Per National Family Health Survey (NFHS)-5 (2019‐21), the median age at first marriage among men aged 25‐29 years nationally was 24.9 years [37,38]. Beyond this age, men’s menstrual knowledge increasingly reflects spousal rather than natal household influence.

Sample size rationale draws on the concept of information power of Malterud et al [39], whereby the number of participants needed decreases as the relevance and richness of the information held by the sample increases. Based on this concept of information power and ethnographic recommendations for an exploratory and flexible study design, we aim to recruit approximately a total of 18‐28 informants from 5‐8 households. The final sample size will depend on when redundancy is confirmed. Participants will be recruited to take part in four separate, homogenous FGDs of 5‐8 participants each, conducted separately with adolescent girls, their mothers, fathers, and adolescent brothers, in order to foster a peer-group environment and reduce discomfort across age and gender lines. IDIs will be conducted with a subset of FGD participants, selected based on willingness to discuss personal menstrual experiences, whole-household participation (adolescent girl who menstruates, mother, father, and preferably an adolescent brother), and responses during FGDs that highlight critical aspects of participants’ physical, social, or enabling environment. The breakdown of the sample size for each informant category is presented in Table 1.

**Table 1.** Study sample.

Research informant category	Objective to interview the informant	Number of informants^a^ (N=18-28)
Adolescent girls who menstruate: Novice	To understand their menstrual experiences and how they perceive of gender, culture, morality, and politics shape their experiences. To understand how they respond to the menstrual environment including norms, restrictions, access to period products like sanitary pads, WASH^b^ infrastructure, menstrual literacy, social support from the family, friends, and community.	5‐8
Adolescent girls who menstruate: Experienced	To understand their menstrual experiences and how they perceive of gender, culture, morality, and politics shape their experiences. To understand how they respond to the menstrual environment including norms, restrictions, access to period products like sanitary pads, WASH infrastructure, menstrual literacy, social support from the family, friends, and community.	5‐8
Mothers of adolescent girls	To understand how menstrual knowledge is transferred down across generations to shape menstrual practices and experiences.	5‐8
Fathers of adolescent girls	To understand their menstrual awareness and the role of gender and gendered norms shaping menstrual experiences. To understand how gender impacts one’s understanding of menstruation and its related practices, beliefs, and ultimately menstrual experiences.	5‐8
Male adolescent siblings of adolescent girls	To understand their menstrual awareness and the role of gender and gendered norms shaping menstrual experiences. To understand how gender impacts one’s understanding of menstruation and its related practices, beliefs, and ultimately menstrual experiences.	5‐8

a Total households: 5-8.

b WASH: water, sanitation, and hygiene.

Data collection will conclude based on data saturation or redundancy across both FGD and IDI data, defined as recurring responses and similar experiences being shared across participants with no new themes emerging. Redundancy is assessed on an ongoing basis through field notes completed after each field visit and verified periodically by reviewing interview audio recordings after every interview. This data saturation assessment will be discussed and validated by the primary author’s doctoral supervisory committee verbally.

### Data Generation Procedures

#### Overview

In CE, data can be a person, a group, documents, or any artifact that holds cultural meaning. In alignment with the methodological recommendation of CE and guided by our theoretical approaches, we are using the following data generation strategies to answer the research questions (Figure 1)

**Figure 1.**
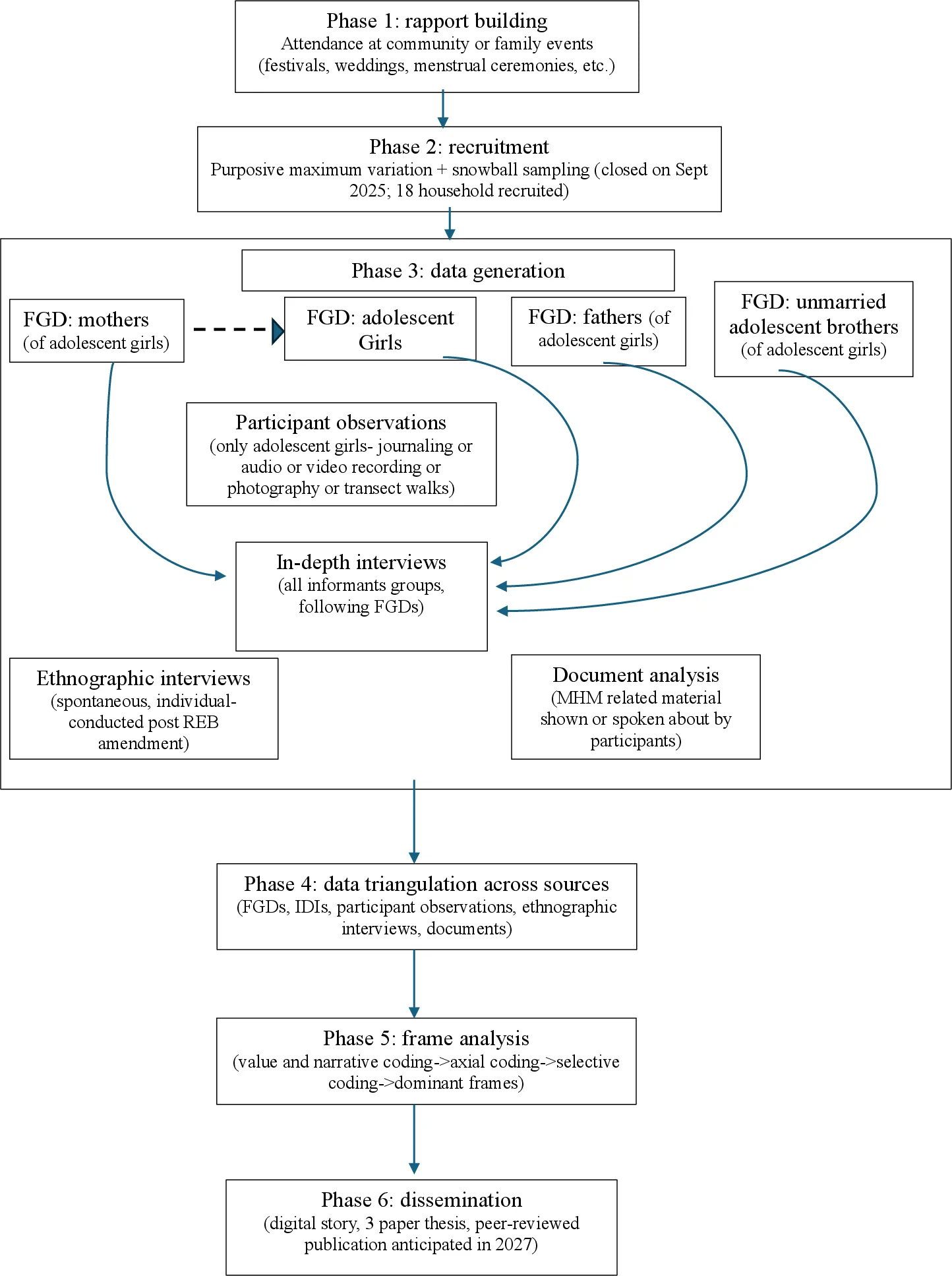
Study design and phases of data collection, analysis, and dissemination. FGD: focus group discussions; IDI: in-depth interviews; MHM, menstrual hygiene management; REB: Research Ethics Board.

#### Rapport Building

This will entail building trusting relationships with informants, explaining the study and its implications, and describing our roles as researchers. Some of the rapport-building activities include attending some of the family and community events, such as festivals, events at places of worship, birthdays, weddings, and menstrual ceremonies. Menstrual ceremonies are cultural rituals marking a girl’s first menstruation as a celebratory rite of passage into womanhood, known locally in the Telugu-speaking region as Ritu Kala Samskara, or the “half-saree function,” in which the girl is dressed in new attire and formally presented to family and community with gifts and blessings [24,40].

#### Participant Observation

To account for the sensitive nature of the topic and varying literacy levels, we are using multiple participant observation strategies based on the comfort of the informants. Informants can choose to record or journal their menstrual experiences for a month in a book, voice records on their phone, or take pictures of the environment around them that speak to their menstrual experiences. If informants are not comfortable with journaling, they can choose to engage in a transect walk [34] with the lead researcher in their community to discuss why and how they use specific spaces in their homes and community during their period and how these spaces define their menstrual experiences (Multimedia Appendix 1). During these observations and interactions, if the informants show or share any document that is relevant to MHM, we will review them to inform them of FGDs and IDIs as needed. We will observe additional locations based on participant input and accessibility to the sites. These locations may include pharmacies, pad disposal sites, and workshops on sexual and menstrual health organized by schools, government agencies, or NGOs to understand their impact on menstrual experiences.

#### Document Analysis

During our observations and interactions, if the informants share relevant documents like brochures, infographics, and pamphlets that are relevant to MHM, we will review them to guide the FGDs and IDIs as needed. Document analysis will only be conducted to contextualize participant responses and as a means of triangulating data from other data sources. Documents will be reviewed in their entirety to compare the participants’ responses with the objectives of the document to understand how the documents align with the participants’ experiences and how their perceptions of these documents align.

#### FGDs and IDIs With Adolescent Girls and Their Families

The purpose of using FGDs and IDIs is to first understand common sociocultural practices, gender, power structures, and other sociopolitical and geographical factors that shape menstrual experiences in slums. The responses from the FGDs will then serve as probes for IDIs to clarify how individual menstrual practices and experiences compare with standard ideologies and expectations.

We will conduct FGDs, followed by IDIs with each of the following informant groups separately: (1) adolescent girls who recently reached menarche or novice menstruators, (2) experienced menstruators, (3) mothers, (4) fathers, and (5) unmarried older brothers (15‐24 years old). These discussions and interviews will likely each take 60‐90 minutes. Informants for IDIs will be selected from each of these informant groups based on the informant’s willingness to participate, availability, experiences, and responses during FGDs.

#### FGDs and IDIs With Menstruators and Their Families

The purpose of using FGDs and IDIs is to first understand common sociocultural practices, gender, power structures, and other sociopolitical and geographical factors that shape menstrual experiences in slums. The responses from the FGDs will then serve as probes for IDIs to clarify how individual menstrual practices and experiences compare with standard ideologies and expectations. We will conduct FGDs, followed by IDIs with each of the following informant groups separately: (1) novice menstruators (1‐2 years since menarche), (2) experienced menstruators (3 years or more since menarche and unmarried), (3) mothers, (4) fathers, and (5) unmarried older brothers (15‐24 years old). These discussions and interviews will likely each take 60‐90 minutes. Informants for IDIs will be selected from each of these informant groups based on the informant’s willingness to participate, availability, experiences, and responses during FGDs (Multimedia Appendix 1).

FGDs will be conducted via a participatory activity and complete the story [41,42]. During these discussions, informants will be asked to complete a fictional story about an adolescent girl who menstruates and their experiences in a slum such as theirs. This story completion technique was used to initiate and generate discussion, given the culturally sensitive nature of menstruation and the likelihood that some participants would be reluctant to discuss their own personal experiences directly. Responding to a fictional, third-person scenario would allow participants to indirectly express norms, beliefs, and practices around menstruation without requiring personal disclosure

#### IDIs With Adolescent Girls who Menstruate and Their Family Members

The purpose of IDIs is to offer the informants an opportunity to safely discuss their personal menstrual experiences and explore how they compare their responses to the vignette shared as part of the complete story activity.

### Ethical Considerations

#### Ethics and Data Management

Institutional ethics approval was secured from the University of Toronto (RIS Human Protocol Number: 46162) and the University of Hyderabad (IEC Number: UH/IEC/2024/221). All participants will receive a comprehensive explanation of the study, including their right to withdraw at any stage without consequence. Based on the comfort of the participants, we will obtain either written or verbal consent from adults and assent from minors (accompanied by parental permission). To ensure full comprehension, translated forms will be read aloud to participants, followed by a question and answer session, after which they will receive a copy of the signed forms. Specific consent will also be sought to use anonymized responses from observations and interviews for the digital story. To maintain confidentiality, data will be stored on password-protected servers at the University of Toronto using unique, randomly generated identifiers. Access is restricted strictly to the research team. Data will be retained for approximately 3 years or until results are published, after which it will be destroyed according to institutional protocols.

#### Compensation

Participating households are eligible to receive up to 1000 INR (~15.40 CAD; CAD $1= US $0.72 as of January 15, 2026) as compensation for their time. This amount is allocated individually: 400 INR each for the mother and father, and 100 INR each for the adolescent girl and her adolescent brother. Compensation is structured on an individual basis; therefore, a participant remains eligible for payment even if other household members withdraw. Compensation is paid as a single amount at the conclusion of each participant’s involvement in the study, based on their level of completed participation. The amount allocated to the adolescent girl and her adolescent brother is not paid to them directly; it is given to the mother, at the community’s suggestion, as a gesture of appreciation for permitting her children’s participation to minimize any risk of misuse of funds by a minor. This amount was kept deliberately minimal and was benchmarked against ethical guidance on avoiding undue inducement in the compensation of minors, rather than the daily wage–based benchmarking used to determine adult compensation amounts.

### Data Generation

Interviews with adolescent girls will be conducted in either their homes, local government–funded school premises, or in another place that is deemed safe and convenient for the informant and our research team. The interviews will be conducted with informant consent either in Telugu, Hindi, or English, based on the informant’s preference and with the support of two trained research assistants, a male and a female, from the University of Hyderabad, India, who are fluent in these languages. These interviews will be audio recorded upon consent; if not, manual notes will be taken. To be respectful and sensitive to the community’s sentiments and cultural norms, the female research assistants will interact with female informants, and the male research assistant with the informants. We will use field notes to record our reflections during and after participant observations and interviews. The primary author and study research assistants will take a collaborative approach to the data generation process where we come together at the end of each day, after our interactions with the informants, debrief on our notes and experiences, and make necessary changes to our data generation approaches.

### Data Analysis

We will adopt an iterative approach that moves back and forth between inductive and deductive analysis, guided by frame analysis [43]. This analysis is both data-driven and theory-driven: frames are initially generated inductively from participants’ own language and observed practices and are subsequently refined and interpreted deductively through the theoretical lenses of biopolitics and post-structuralist feminism. A “frame” refers to the underlying lens or storyline that shapes how a person understands and describes their experience. Frame analysis looks at both what people say and how they say it, in order to understand how individuals, media, or institutions construct and present a version of reality. In this study, we use this approach to uncover the unspoken assumptions and power dynamics that shape how menstruation is understood and experienced in the study communities.

The authors will begin by using empirical observations from the study site to identify patterns relevant to the research topic. These patterns are then explored further and tested against what participants say directly in interviews.

All audio recordings will first be transcribed in full to allow for a close, detailed reading of the data. As the sole researcher conducting this analysis, a reflexive journal will be maintained by the first author (SV) throughout the coding process to document analytic decisions, initial impressions, and potential sources of interpretive bias, and will periodically revisit earlier-coded transcripts to check the consistency of code application as the coding framework develops. The transcribed interview data, along with data from participant observations, will then undergo an initial round of coding, meaning the data is broken down into small, labeled segments that capture key ideas. The authors will use value coding to label data according to the values, beliefs, and attitudes participants express, and narrative coding to label data according to the structure and content of the stories participants tell [44]. This initial coding stage helps identify the dominant and recurring frames present across the data. Codes and emerging frames identified at this stage are data-driven, arising inductively from participants’ own words and observed practices rather than being imposed from existing theory.

Next, the authors will use axial coding [43], meaning they look for connections between the codes identified in the first stage and group related ideas into broader categories, referred to as discourses. From these broader categories, the authors identify recurring underlying assumptions, or ideologies, and examine them further through selective coding [44], a process of refining and narrowing the analysis to uncover the deeper ideological foundations of these assumptions. This step is guided by existing literature, relevant documents, and theoretical perspectives such as biopolitics and poststructuralist feminism, both of which are concerned with how power and social norms shape people’s lived experiences. This stage introduces a deductive element to the analysis, as emerging discourses and ideologies are examined against, and interpreted through, these established theoretical frameworks.

Finally, these underlying ideologies are synthesized into a smaller number of dominant frames. A frame is considered “dominant” if it recurs across multiple informant groups and data sources (FGDs, IDIs, and participant observation) and is validated through triangulation across these sources; frames that are not corroborated across multiple sources or informant groups are treated as context-specific rather than dominant. These frames illustrate how adolescent girls in informal settlements (bastis) experience menstruation and highlight the social norms and power structures that shape these experiences, pointing to areas where change may be possible.

Given that coding will be conducted by a single researcher (SV), analytic rigor will be supported through several strategies rather than intercoder agreement: (1) a detailed coding log and reflexive journal will be maintained to document how codes and frames evolve and to provide an audit trail of analytic decisions and (2) preliminary frames and interpretations will be periodically discussed with the dissertation committee and research supervisors to test their plausibility and guard against idiosyncratic interpretation. While emerging interpretations will not be formally member-checked with participants during the analysis process itself, final study results will be shared with participants as part of the digital story output described in the Knowledge Mobilization and Dissemination section, offering an opportunity for participants to engage with and respond to the study’s findings following analysis.

Although research questions 1 through 3 address conceptually distinct dimensions of menstrual experience, sociocultural and political discourse, intergenerational household power dynamics, and the enabling environment, these are treated as equally weighted, interconnected dimensions of a single, holistic phenomenon rather than three independent lines of inquiry requiring separate analytic capacity. The same participants, households, and interactions generate data relevant to all 3 research questions simultaneously; for example, a single interview or observation may reveal a participant’s sociopolitical framing of menstruation, the household power dynamics shaping her experience, and the material conditions of her enabling environment, all at once. This reflects the strength of an ethnographic approach, which is well suited to exploring multiple, interconnected dimensions of a bounded community in depth, rather than treating each dimension as a separate object of study.

Data relevant to each research question are therefore not isolated at any single coding stage; instead, the dominant frames that emerge from the full analytic process are subsequently examined and categorized according to their relevance to each research question, guided by the theoretical frameworks underpinning this study. Research question 1 is interpreted jointly through both biopolitics and poststructuralist feminism, examining how institutionally produced discourses around menstrual hygiene are perceived and absorbed by adolescent girls themselves. Research question 2 is interpreted primarily through poststructuralist feminism, attending to the diffuse operation of power within intergenerational households through inherited norms and discourse. Research question 3 is interpreted primarily through biopolitics, treating the material and infrastructural conditions of the enabling environment as themselves a product of institutional governance. In this way, the theoretical frameworks guide the interpretation of findings in relation to each research question, once these findings have emerged from the complete frame analysis process. The expanded scope of this study, including engagement with local NGOs, a community health worker, and a school teacher frequently referenced by participants, further strengthens the depth of data available to address research questions 1 and 3, given their relevance to sociopolitical discourse and the structural enabling environment respectively.

## Results

### Overview

This study is funded by the International Development Research Centre (IDRC) through the International Doctoral Research Award (2024‐2025). This study has received ethics approval from the University of Toronto Research Ethics Board and the institutional ethics committee at the University of Hyderabad, India in January 2025. By September 2025, the authors have recruited 18 households including 11 adolescent girls, 18 mothers, 4 fathers, and 1 brother. In the process of building rapport and getting to know the community during the initial stages, the lead author (SV) also met with the local community health workers, a high school science teacher who works at the local public school, a doctor at the urban health center situated in the community, and the founder of an NGO who was highly spoken of by the community members. In terms of baseline characteristics, the sample reflected the following profile: adolescent girls aged between 9 and 17 years old, and therefore assent was obtained from them post parental consent. Most of the fathers either worked as day laborers or security personnel. Mothers were most employed as maids or housekeeping staff in hospitals or shopping malls. The dominant religions were Hinduism and Christianity.

Data collection was closed in March 2026. The majority of data collection occurred between April 2025 and January 2026. Triangulation activities, involving follow-up questions based on the initial review of interview audio, concluded in March 2026. Study findings are anticipated to be disseminated and published by September 2027. The complete study timeline is presented in Table 2.

Several modifications were made to the original research plan during fieldwork, ranging from changes in study scope to refinements in data collection methods and consent procedures.

**Table 2.** Study timeline.

Phase	Timeframe	Key activities
Planning and preparation	March 2024-January 2025	Ethics approvals, recruitment and interview guides, hiring research assistants
Rapport building	February 2025-March 2025	Familiarizing with the community and other key informants in the community
Participant recruitment	March 2025-September 2025	Consent and assent were obtained from potential participants once the research study was explained to them.
Data Collection	April 2025-March 2026	Generating data through participant observations, focus group discussions, and in-depth interviews.
Data analysis	May 2026-November 2026	An iterative-inductive and deductive approach via frame analysis
Knowledge translation and mobilization	April 2026-September 2027	Digital story, a three-paper model of a doctoral thesis, and peer-reviewed publications.

### Scaling Down the Study Scope

The authors had originally designed the study to be conducted across 5 slum colonies within the broader Film Nagar slums, which comprise 18 colonies in total. After initiating conversations with a few families in each, it became clear that meaningful rapport-building across all 5 sites would require an additional 6 months of fieldwork, as ethnographic data collection depends on sustained and visible presence within each community. Given the time constraints of the doctoral program and the IDRC award period, they scaled the study down to two colonies—MG Nagar and Patel Nagar—that proved most accessible and where we could undertake rapport-building with sufficient depth.

### Sequencing Methods to Build Trust

Our initial plan to begin participant observation directly with adolescent girls in MG Nagar was complicated by parental mistrust about the study’s purpose. The authors adapted the sequencing of methods to begin with FGDs with mothers as a sensitization strategy, which helped clarify the research intent and build trust before the authors engaged with their daughters who reached menarche. This shift did not alter the research questions but reshaped the emphasis of methods, with mothers becoming an important constituency in their own right rather than purely a gatekeeping group.

### From Large FGDs to Small Group Discussions

Initial FGDs in the community hall consistently drew only 2 participants, as women preferred to return home directly after long workdays and were reluctant to walk to the hall. Following consultation with the supervisory committee, the authors submitted and received approval for an REB amendment to include small group discussions of 2-3 participants. We also relocated sessions from the community hall to participants’ homes—typically anchored in 2 trusted households per lane—on the recommendation of a community contact who noted that Sundays were guarded as private family time.

### Adding Ethnographic Interviews

When even small group sessions sometimes produced only a single willing participant, we submitted a further REB amendment to formally include ethnographic interviews generated through spontaneous individual interactions. This expanded our capacity to engage women with irregular schedules and individuals who approached me independently.

### From Written-Only to Written and Verbal Consent

We obtained written consent without difficulty in the early stages of fieldwork. However, several women later declined to sign documentation, citing concerns rooted in past experiences and partner objections. A final REB amendment was submitted and approved to permit verbal consent as an alternative to written consent, in keeping with established ethical practice for low-literacy and trust-sensitive research contexts.

### Scheduling and Contact Strategies

Participants’ work schedules, festival cycles (Ugadi, Eid, and Good Friday), the August monsoon, and limitations on mobile phone use among women in households with strained partner relationships required ongoing adaptation of fieldwork timing and modes of contact. Recruitment among Muslim women proved unfeasible despite repeated outreach.

## Discussion

### Anticipated Contributions

We anticipate that our proposed study will make meaningful contributions to the existing research on MHM and sexual and reproductive health (SRH). By uncovering the complex interplay of social, political, environmental, and family power dynamics, our study will provide a vital understanding of the unique elements that affect the menstrual experiences of adolescent girls living in these low-resource and marginalized settings. Such insights are crucial for creating context-specific, equitable, and culturally sensitive programs that effectively serve the needs of adolescent girls in slums and other low-resource settings. Menstruation is a biological phenomenon and “not a woman’s thing” [45]. It is a gendered experience for adolescent girls in various sociocultural domains. Unpacking the social aspects of menstruation through a gendered lens will help understand how gender impacts one’s understanding of menstruation and its related practices, beliefs, and ultimately menstrual experiences. Although the research informants may not incur direct and immediate benefits from this research, findings from our research will play a significant role in designing culturally sensitive, participant-informed interventions and policies to improve the menstrual experiences of adolescent girls living in similar informal urban settlements.

### Knowledge Mobilization and Dissemination

To ensure that the findings of this study reach a wide range of audiences, we disseminate the findings in 3 formats: a digital story, a 3-paper model of a doctoral thesis, and stand-alone papers will be submitted to peer review publications.

Post analysis we will select 4‐5 stories to develop a 3‐ to 5-minute digital story [46,47] that depicts the essence of menstrual experiences in slums and portrays participants’ recommendations to improve them. This digital story will first be pilot tested with the research informants and incorporate their feedback before making the digital story public. This participatory approach will allow the research informants to play an active role in determining the relevance and appropriateness of the stories shared to communicate study findings.

### Limitations and Challenges

We anticipate potential challenges that may affect our study’s implementation, particularly regarding the lead researcher’s positionality, study scope, and time constraints. The lead researcher’s (SV) identity as an Indian citizen studying in a Canadian academic institution and her affiliation with the Ishva Foundation could influence how participants perceive her or share their experiences regarding sensitive topics like menstruation. To mitigate potential hesitation and navigate these power dynamics, the lead author (SV) will rely on community gatekeepers to build trust. The author plans to collaborate with established community figures—including Accredited Social Health Activists (ASHA) who are trained female community health volunteers, local basti leaders, and my former students residing in the community—who will act as cultural bridges and liaisons during the recruitment and data generation phases.

The focus of this study is specifically on understanding the perspectives of adolescent girls, allowing us to examine how sociocultural, spatial, and institutional contexts shape their experiences. However, it is important to note that these perspectives might diverge from those of health care providers or policymakers. We intend to address this gap in future research.

Another significant challenge is participant reluctance, particularly among men and individuals from conservative households, to engage in discussions about menstruation. This reluctance could further complicate data generation. Additionally, the migratory and transient nature of the slum population presents obstacles for follow-up, making comprehensive data gathering more difficult.

Moreover, all ethnographic studies face inherent time constraints. Gaining an in-depth understanding of lived experiences requires substantial time investment, and our study’s scope is limited by the funding and scheduling demands typical of doctoral programs. Despite these limitations and challenges, this study prioritizes contextual awareness, reflexivity, and lived experiences to ensure a significant contribution to the field of menstrual health research.

In conclusion, this study protocol outlines a path towards contextual and gender-sensitive understanding of menstrual experiences in marginalized communities. It is our expectation that the insights disseminated through this word will lay the foundation for sustainable, participant-informed solutions that effectively improve the quality of life for adolescent girls in urban slums.

## Supplementary material

10.2196/94012 Multimedia Appendix 1 Data collection instruments.

10.2196/94012 Checklist 1 Standards for Reporting Qualitative Research (SRQR) checklist.

## Abbreviations

ASHA: Accredited Social Health Activists
CE: critical ethnography
FGD: focus group discussion
IDI: in-depth interview
IDRC: International Development Research Centre
MHM: menstrual hygiene management
NFHS: National Family Health Survey
NGMHM: National Guidelines for Menstrual Hygiene Management
NGO: nongovernment organization
REB: Research Ethics Board
SRH: sexual reproductive health
SSHRC: Social Sciences and Humanities Research Council
WASH: water sanitation and hygiene
WSSCC: Water Supply and Sanitation Collaboration Council
