# Supplementary material for: Uncovering the Experiences of Basti-Dwelling Adolescent Girls Who Menstruate in Hyderabad, India: Protocol for a Multimethod Critical Qualitative Study
Source: JMIR Res Protoc. 2026 Oct 19;15:e94012. doi: 10.2196/94012 (PMC13679868; doi:10.2196/94012)
Supplement: Supplementary file 2 [file resprot-v15-e94012-s001.docx]

**Focus Group Discussion-Complete-the-story: Vignettes, questions and probes.**

**Objective:** To discuss, reflect, and understand the menstrual culture and practices at play the community at large.

**Purpose of the group discussion:** The purpose of this group discussion is to have an open and non-judgmental discussion about what it is like to be a menstruator in this basti. I want to understand about the general menstrual practices and norms that families in this basti follow. Through this discussion group I would like to learn about some positive aspects of this basti that help menstruators feel safe and comfortable during their period. I would also like to know how the community spaces could be improved to ensure menstruators have a positive menstrual experience.

Upon your consent, this discussion will be recorded for analysis purposes and deleted by the end of the study. Please note that participation in the study is voluntary. You can refuse to participate, withdraw from the study, or decline to answer any question or participate in any part of the study with no consequences.

**Participant ID:**

**Name of the interviewer Participant’s**

**age:**

**Participant’s gender:**

**Participant’s educational status:**

**Participant’s Employment status:**

**Participant’s marital status:**

**Participant’s religious affiliation:**

**Participant’s caste affiliation:**

**Estimated time: 60-90 minutes Location:**

**Date:**

**Start time:**

**End time:**

**************************************************************************** Introduction**

Hello, thank you for being willing to spend your time with me to share your knowledge and experiences with menstruation.

I will now share a story of a young girl, Amala, who lives in a similar community to yours and belongs to a similar family background as you. I will share this story in parts followed by a few questions, and I want you to answer them as if you were Amala to complete the story. There might be incidents where you answer the question as if Amala is your friend and provide her with recommendations based on your experiences.

***************************************************************************

**Socio-cultural Influences on Menstruation**

**Vignette:** Amala is a 12-year-old girl who lives in a nearby slum. She is very playful and goes to the local government school. She also has an older brother, Arjun. He is 15 years old and attends the same school as Amala. One day, when she went to bathe in the morning, she noticed blood in her underwear.

I now want you to answer a few questions for this part of the story:

| **Q.no.** | **Interview questions** | **Probes** |
| --- | --- | --- |
| 1 | What do you think happened to Amala? Why is  there blood in her underwear? |  |
| 2 | How do you think Amala reacted when she saw the blood? | 1. What do you think she would have done next? 2. Whom do you think she would have called or is it appropriate to call for to share about this incident? 3. What do you think they would have done? 4. What do you think they would have told her? 5. What material/product do you think Amala will be asked to use during her period? Why? 6. In general when are girls told about period and what is told to them? Who gives them this information? What about the   boys in the family? |
| 3 | How do you think Amala’s life will change  from here on? | 1. Compared to her brother   (Think in terms of changes in her social engagements,  educational pursuits, and marriage).   2. How will Amala’s period   affect her attending school?   3. What will Arjun, Amala’s brother be told about what happened to Amala? 4. Will anything change in terms of:    1. Places she visits    2. People she speaks to    3. What she eats    4. What she wears    5. Her relationship and interactions with people in the family and the community? 5. Are there any common traditions followed when a girl first menstruates? What are they? 6. How did these traditions come about? How important is it for |

|  |  | your daughter to follow them?   7. Have any of these traditions changed when one converts to a new religion? Why? 8. Are there any differences in how these traditions are followed in your villages vs in the basti? How are they different? Why? When   you visit your village, which tradition/norms do you follow?   9. How would you say the menstrual   practices and norms that your followed when you were your  daughter’s age differ from hers? What’s caused that shift? |
| --- | --- | --- |

**Vignette continues:** It's been 5 years since Amala first got her period. She seems to have gotten used to a monthly routine during the 3-7 days of her periods? She has been experiencing some changes in her body and seems to also have health issues related to her periods

| **Q.no** | **Interview questions** | **Probes** |
| --- | --- | --- |

| 3 | How do you think Amala deals with her period and related bodily changes or any other period related issues like pain or missed periods? | Think in terms of who she trusts or is acceptable to reach out to when she’s faced with menstruation related issues.  As a parent how do you deal with such situations? (do you go by traditional knowledge or ask a friend or family member or reach out to a doctor, or ask about it at an NGO led  workshop?) |
| --- | --- | --- |

**Vignette continues:** Amala gets her period when her mother is away from home and realizes she does not have a sanitary pad or cloth. Her father and brother are at home.

| **Q.no** | **Interview questions** | **Probes** |
| --- | --- | --- |
| 4 | What do you think Amala will do? | Do you think she’ll ask her dad or brother to get her a pad? If they respond no, ask why not? |
| 5 | How do you think Arjun and/or their father can support Amala during her period? | Do you think it would be acceptable in her family to talk to her father or brother about her period and her related needs like certain menstrual hygiene products, access to private toilet, menstrual pain management  medications, doctor visits, etc.? |

**Communal Tensions and Precarious Lifestyles**

**Vignette Continues:** Amala lives in a slum like yours and attends the local government school. Amala lives in a small, one-room home with no separation for the kitchen, bedroom and dining area. She and her family do not have a private bathroom and toilet. They use a shared bathroom and toilet with four other households. Amala’s household also has no separate backyard or balcony to wash and dry their clothes. They use a shared wash and dry area with four other neighbours.

| **Q.no** | **Interview questions** | **Probes** |
| --- | --- | --- |
| 6 | What does it mean for girls like Amala who lives in the slums to have her period? | Based on your personal experience, what are some of the common challenges Amala might face with managing her periods in a slum like yours? including when she is at school.  What are some suggestions you have for Amala to ensure she has safe and comfortable periods?  Please think in terms of the menstrual care product she uses, how she washes, dries, and disposes of her pads, where and when she can change, avoid unnecessary attention, people she could talk to about her period, how she can clean herself when menstruating, etc.  From your experience, what are some special safety measures Amala should take to ensure her personal safety in the community because she her new menstrual status? |
| 7 | How would you like your community (including the school environment) to change to ensure girls like Amala can have a safe and comfortable  period? | Think in terms of safe places to change, disposal and drying menstrual material, water access, toilets, communal violence,  sexual abuse, etc. |

**Influence of Enabling Environment on Menstruation**

**Vignette continues:** One day, Amala comes across an advertisement on period and sees a girl who is active, happy and attends school during her period. The ad shows new menstrual hygiene product (could be a pad, menstrual cups, etc.,) on TV and in NGO-led MHHM workshop in school, and she wants to try them.

| **Q.no.** | **Interview questions** | **Probes** |
| --- | --- | --- |

| 8 | If Amala was your daughter/sister/or relative, what factors will influence her menstrual practices like menstrual hygiene material she uses and her general  lifestyles during her period? | Think in terms of her prospects of continuing school or getting married. |
| --- | --- | --- |

**Vignette continues:** A local NGO led a MHHM workshop about the science behind menstruation, physiological changes that occur due to menstruation, period management strategies using pads, tampons, cups, and cloth. They also debunked some of the most prevalent taboos around menstruation.

| **Q.no** | **Interview Questions** | **Probes** |
| --- | --- | --- |
| 9 | How do you think the menstrual practices and beliefs of the family changed after attending this workshop?  Have you attended any similar workshops? What were you told? Did you learn anything new? What was it? Have you implemented it into your life? | What do you think they will do with this new information? Do you think they will educate their family? How do you think their family would react? or do you think they’ll ignore them and follow their routine?  If they decide to challenge some of the current norms and taboos and not follow the regular norms how would their family react?  If she decides to challenge some of the current norms and taboos and not follow the regular norms she was taught by her family, like participating in religious rituals,  how would her family react? |

**Closing**

| **Q.no.** | **Interview questions** | **Probes** |
| --- | --- | --- |
| 10 | We have come to an end with this interview, how do you feel about our conversation today and is there anything else  you would like to tell me? |  |

**In-Depth Interview Guide**

**Objective of In-depth Interview:**

1. To understand how menstruators and their immediate family perceive of menstruation and its effects on their lives
2. To unpack the diverse factors ranging from socio-cultural norms, gender, politics, familial power dynamics, media and physical environment, that shape menstrual experiences in a *basti* (informal urban settlements or slums)
3. To understand their menstrual awareness and the role of gender and gendered norms shaping menstrual experiences.
4. To understand how gender impacts one’s understanding of menstruation and its related practices, beliefs, and ultimately menstrual experiences.
5. To understand how menstrual knowledge is transferred down across generations to shape menstrual practices and experiences.

**Purpose of the interview:** The purpose of this interview is to discuss your individual experiences as a menstruator or being related to a menstruator as a parent or a sibling are. Through this interview I wish to understand how your menstrual experiences compare to the general norm in the community.

Upon your consent, this discussion will be recorded for analysis purposes and deleted at the end of the study. Please note that participation in the study is voluntary. You can refuse to participate, withdraw from the study, or decline to answer any question or participate in any part of the study with no consequences.

**Participant ID:**

**Name of the interviewer Participant’s age:**

**Participant’s gender:**

**Participant’s educational status:**

**Participant’s Employment status:**

**Participant’s marital status:**

**Participant’s religious affiliation:**

**Participant’s caste affiliation:**

**Estimated time: 60-90 minutes Location:**

**Date:**

**Start time:**

**End time:**

******************************************************************************

**Rapport building:**

“A discussion on periods might not be a preferred topic for everyone. But it is widely recognized as a defining moment in a girl’s life. Having access to the necessary knowledge to care for themselves and make informed choices is crucial for a girl’s well-being and empowerment. Open conversations about menstruation can contribute to better understanding, support, and opportunities for girls, aiding them in achieving their goals and securing a stable, independent future.”

**Introductory questions:**

| S.no. | Questions | Probes |
| --- | --- | --- |
| 1 | For girls: when was the first time you had your period? | what did you know about  period when you first menstruated? |
|  | For mothers and fathers: when was the first time your daughter had her period? | How prepared was your daughter for her first period? |
|  |  | How important do you think it is to prepare girls for their first period? |
|  |  | How important is  menstruation in a girl’s life? |
|  |  | How does the life of a girl change after menarche? |
|  | For brothers: When was the first time your sister had | How much did you know |
|  | her period? | about period before your |
|  |  | sister had her first period? |
|  |  | What new information did |
|  |  | you learn about periods |
|  |  | after your sister had her first |
|  |  | period? |

| 2 | For girls: Can you share some details about what happened in the house when you had your period?  For parents: Can you share some details about what happened in the house when your daughter had her period?  For brothers: Can you share some details about what happened in the house when your sister had her period? | For all: What was your first reaction?  For girls: What was your parent’s first reaction?  For girls: What kind of customs were followed when you first menstruated? For all: What kind of customs are usually followed when a girl reaches menarche?  For parents: What customs did you followed when your daughter first menstruated? For all: how common is it to have a menstruation ceremony in your family?  For girls: did your family  have such a celebration for |
| --- | --- | --- |

|  |  | you? What is the importance of this ceremony? What is done during these ceremonies? Who is called?  For parents: did you have such a celebration for your daughter? What is the importance of this ceremony? What is done during these ceremonies? Who is called?  For brothers: did your family have such a celebration for your sister? What is the importance of this ceremony? What is done during these  ceremonies? Who is called? |
| --- | --- | --- |

**Influence of Socio-cultural factors (norms and power dynamics in families)**

| **S.no** | **Interview questions** | **Probes** |
| --- | --- | --- |
| 1 | **For all:** What do you know about menstruation? | When did you first learn about menstruation?  Can you tell me about how you first learned about menstruation? **For menstruators and brothers**: How did you feel when you first learned about periods?  **For menstruators:** How did you feel when you first had your period? How aware and  prepared for your first period? **For mothers and fathers:** how did it feel when your daughter first menstruated? What were some of the first thoughts that ran in your mind?  **For mothers:** Do you remember what you told your daughter or son about menstruation? When? Was it the first time you spoke to them about it? Was it any different from what you were told when you first menstruated? Explain. **For fathers:** did you ever talk to your daughter or son about  menstruation? When? What did you tell them? |

| 2 | **For menstruators**: In what ways has your life changed since your first period?  **For mothers and fathers**: How has your  daughter’s life changed since her first period?  **For brothers**: How has your sister’s life changed since her first period compared to yours? | How differently are you/is your daughter/your sister treated by your family since you/she started to menstruate compared to your/her brother? Think in terms of what you are allowed to do and not do? This could be places you visit, food, people to speak with in the community?  Has there been any change to how the community you live in perceives/thinks of you/your daughter/your sister? Explain.  **For menstruators**:  How do you feel about being treated differently?  **For family**: Do you know how she feels? Why should she be treated differently?  **For menstruators and brothers:** How differently would you treat your future  daughter when she menstruates? Would you continue to follow the same traditions that your  family follows now? Explain |
| --- | --- | --- |
| 3 | **For menstruators:** How does your family, including your brother and father, support you during your periods?  **For mothers and fathers:** How do you support your daughter during her period?  **For brothers**: How do you support your sister during her period? | **For menstruators and brothers:** Whose responsibility is it to support you/your sister in matters related to your/her  period? Why?  **For mothers and fathers**: Whose responsibility is it to support your daughter in matters  related to her period? Why? |
| 4 | **For menstruators:** How would you describe your lifestyle during the 3-4 days of your period?  **For mothers and fathers:** How would you describe your daughter’s lifestyle during the 3-4 days of your period?  **For brothers:** How would you describe your sister’s lifestyle during the 3-4 days of your  period? | **For menstruators**: What is the kind of rules are you expected to follow during your period?  What are you allowed to do and not do during your periods?  Why? |

|  |  | Are there any places you are allowed to go and not go during your periods? Why?  How did you learn about these rules of a period?  How important is it for you to abide by these rules?  Do you have any issue with these rules? How have they affected your life? Do you think they are necessary and should be followed?  Do you abide by these rules? Is there any flexibility you have or make around these rules? Which ones are they? Explain why or why not you abide by them?  Have you challenged any of these rules?  In what ways have you challenged them and why?  Are there any consequences of breaking these menstrual rules?  **For mothers and fathers:** What is the kind of rules is she expected to follow during your period?  What is she allowed to do and not do during her periods?  Why?  Are there any places she is allowed to go and not go during her periods? Why?  **For mothers:** Are you flexible around any of these rules with her compared to the ones you were expected to follow when you were her age? |
| --- | --- | --- |

|  |  | **For mothers and fathers:** How important is it for you that she abide by these rules?  Do you have any issue with these rules? How have they affected her life? Do you think they are necessary and should be followed?  Are there any consequences of breaking these menstrual rules? Have you experienced these consequences?  **For brothers**: What is the kind of rules is she expected to follow during your period?  What is she allowed to do and not do during her periods?  Why?  Are there any places she is allowed to go and not go during her periods? Why?  Do you have any issue with these rules? How have they affected her life? Do you think they are necessary and should be followed?  Are there any consequences of breaking these menstrual rules? |
| --- | --- | --- |
| 5 | **For menstruators**: What do you use for managing your period blood?  **For mothers and fathers:** What does your daughter use for managing her period blood?  **For brothers:** What does your sister use for managing her period blood? | **For menstruators:** Is it what your mother uses to manage their period? If not, what is it?  Where have you learned this method of period management?  Is it your preferred method or enforced by your family’s preference and affordability?  If not a preferred choice, why  don’t you shift to your preferred method? |

|  |  | What are some factors that will help or stop you from shifting to your preferred method?  **For mothe**rs: Is it the same as what you use to manage your period? If not, what is it?  Is it her preferred method or enforced by your family’s preference and affordability?  If not a preferred choice, why  doesn’t she shift to her preferred method?  What are some factors that will help or stop your daughter from shifting to your preferred  method? |
| --- | --- | --- |

**Influence of Familial Power differentials**

| **S.no** | **Interview questions** | **Probes** |
| --- | --- | --- |
| 6 | **For all:** What are the different factors that influence the menstrual practices of your family? | What has a greater influence-family traditions, education, family’s financial status, or a specific person like a  grandparent or the head of the family, say father? |

**Influence of Enabling Environment (Politics)**

| **S.no** | **Interview questions** | **Probes** |
| --- | --- | --- |
| 7 | **For All:** How do you think you/your daughter/your sister are supported during your period to care for your menstrual needs and health outside of their home? | Think in terms of WASH infrastructure in the community, safety, school environment, etc.  **For All**: Whose responsibility do you think it is outside your family to ensure you/your daughter have all the support |

|  |  | and resources to manage your period in safety and comfort?  **For All:** Do you feel the government has any role in supporting your/your  daughter’s/ sister’s menstrual needs?  **For All:** How do you think the government can support you/your daughter/your sister in addressing any menstrual  related problems you/she might be facing? |
| --- | --- | --- |

**Influence of Enabling Environment** (**MHHM Workshops**)

| **S.n**  **o** | **Interview questions** | **Probes** |
| --- | --- | --- |
| 8 | **For All**: Have you ever attended any MHM related workshops? If yes, can you please describe what was discussed in the workshop? Did anyone go with you? | **For All:** Can you tell me who conducted and led the workshop and where it was conducted?  How often was this workshop conducted? Why did you attend this session?  Did someone ask  you to? What did you think of the workshop? Did you learn anything new that you didn’t know before? What  was it? |
| 9 | **For All:** How have your menstrual practices or beliefs changed after attending this workshop? | **For Menstruators**:  If not, why?  If you wish to change, what do you need to make that change? What is stopping  you from changing? |
| 10 | **For Menstruators:** How have your family’s menstrual beliefs and practices changed after attending this workshop? | **For All:**  If not, why? |

|  |  | How do you wish them to change to support you to have a positive menstrual experience?  If you wish them to change, what do you think will help them change? What is stopping them from  changing? |
| --- | --- | --- |
| 11 | **For menstruators and brothers:** How has your school influenced your menstrual knowledge,  practices and overall menstrual experience? | How do you think your school could help girls like  you to be better prepared for  their period? |

**Influence of Enabling Environment (Media environment**)

| **S.no** | **Interview questions** | **Probes** |
| --- | --- | --- |
| 12 | **For All:** Have you ever watched an ad/movie related to periods? If yes, can you briefly describe the ad/movie for me? how do you feel about the message shared about menstruation in these ads/movies? | **For all :** What were some similarities or differences you saw with your/your daughter’s own menstrual experiences?  **For menstruators:** how about your family? How do they react to such ads and movie**s** |
| 13 | **For all** How have your menstrual beliefs and practices changed after seeing the ad or movies? | **For All:** If not, why? what is stopping you? What is the role of your family’s influence on your ability to make the changes you wish to make. |
| 14 | **For All:** Is there anything about the ad or movie you would like to change? What would you  change and why? |  |

**Influence of Influence of Enabling Environment (Physical Environment**)

| **S.no** | **Interview Questions** | **Probes** |
| --- | --- | --- |
| 15 | **For All**: What are some common problems or challenges that you and your family face in the slums since you/your daughter began to  menstruate or during your/her periods? | **For All:** Can you describe a time you or your family  member were faced with this challenge? |
| 16 | **For All:** How can the communal spaces including school environment and resources be improved to help local menstruators like you/your daughter/your sister to manage their period with comfort and safety? | **For All:** Who do you think can help address these issues?  Have you reached out to someone to address these  issues? Who did you reach out to? What did they say? |

**Closing**

| **Q.no.** | **Interview questions** |
| --- | --- |
| 17 | We have come to an end with this interview, how do you feel about our conversation  today and is there anything else you would like to tell me? |

# Participant Guide to Participant Observation – Journaling and Transect Walks.

**Purpose of Study:**

My study aims to learn about what it’s like to be a menstruator in a slum and what aspects of your household and the community menstrual experiences are shaped in a basti. Learning from your lived experiences here in the *basti,* will help sound alarm to the urgent needs of menstruators in the community and other similar communities and improve their health, safety, academic and career prospects.

# Purpose of Journaling:

The purpose of this journaling activity is to obtain an idea of your life during your

period. Your journal notes will help me understand what helps and does not help you during your period in your household and your community. You are also welcome to share your

recommendation to improve your menstrual experience.

# Types of journaling strategies to choose from:

1) Written journal- hand-written notes in a physical dairy,
2) Audio journal- voice recorded journals using either their phones or a voice-recorder,
3) Photo journal- take picture of the spaces and things that help explain your menstrual experience.

If you are not comfortable with any of the above journaling strategies, you have the option of participating in a transect walk with me:

4) Transect walks- walk through pre-determined spaces and routes along with me to discuss your menstrual experiences in relation to these specific spaces.

**When to journal:** during the 5-7 days of your current or just past period.

# What to journal:

1) First identify and inform me of the type of journaling strategy you wish to take up.
2) You can record any information the pertains to your period except the faces of any individuals including your family members.
3) To start with you can discuss the following points in your journal:
   a. What do you do on your first day of your period?
   b. Are your expected to follow any specific norms or practices? What are they?
   c. What are some activities you are allowed and not allowed to do?
   d. Who do you inform in the household and why?
   e. What kind of material you use to manage your period?
   f. How do you dispose or clean your menstrual management products?
4) Please note that you are not restricted to answering these questions and can always go beyond these questions to journal about any aspect of your house or the community the impacts your menstrual experiences.
5) At the end of the journaling activity, you will submit your journal entries to me.
6) I will review them and contact you if I have any clarifying questions. If you have chosen to the option of photo journaling, I will meet with you on a separate day to discuss and take notes about the photographs you have taken.
7) If need be, I will ask you to show me certain spaces in your home or the community that you might have mentioned in your journal entry. This is to gain a better understanding of your experiences.
8) Please note that you can always contact me with question about the journaling activity.
